# Public funding of health at the district level in Indonesia after decentralization – sources, flows and contradictions

**DOI:** 10.1186/1478-4505-7-5

**Published:** 2009-04-16

**Authors:** Peter Heywood, Nida P Harahap

**Affiliations:** 1 Australian Health Policy Institute, University of Sydney, Sydney, NSW, Australia; 2 Jalan Bukit Dago Selatan, Bandung, West Java Province, Indonesia

## Abstract

**Background:**

During the Suharto era public funding of health in Indonesia was low and the health services were tightly controlled by the central government; district health staff had practically no discretion over expenditure. Following the downfall of President Suharto there was a radical political, administrative and fiscal decentralization with delivery of services becoming the responsibility of district governments. In addition, public funding for health services more than doubled between 2001 and 2006. It was widely expected that services would improve as district governments now had both more adequate funds and the responsibility for services. To date there has been little improvement in services. Understanding why services have not improved requires careful study of what is happening at the district level.

**Methods:**

We collected information on public expenditure on health services for the fiscal year 2006 in 15 districts in Java, Indonesia from the district health offices and district hospitals. Data obtained in the districts were collected by three teams, one for each province. Information on district government revenues were obtained from district public expenditure databases maintained by the World Bank using data from the Ministry of Finance.

**Results:**

The public expenditure information collected in 15 districts as part of this study indicates district governments are reliant on the central government for as much as 90% of their revenue; that approximately half public expenditure on health is at the district level; that at least 40% of district level public expenditure on health is for personnel, almost all of them permanent civil servants; and that districts may have discretion over less than one-third of district public expenditure on health; the extent of discretion over spending is much higher in district hospitals than in the district health office and health centers. There is considerable variation between districts.

**Conclusion:**

In contrast to the promise of decentralization there has been little increase in the potential for discretion at the district level in managing public funds for health – this is likely to be an important reason for the lack of improvement in publicly funded health services. Key decisions about money are still made by the central government, and no one is held accountable for the performance of the sector – the district blames the center and the central ministries (and their ministers) are not accountable to district populations.

## Background

Following the downfall of President Suharto in 1998, Indonesia underwent a radical political, administrative and fiscal decentralization [1]. Under the new laws and regulations local governments have responsibility for delivery of services in a number of sectors including health. In practice, the actual functional responsibilities of the various levels of government are still unclear. What is clear, nevertheless, is that districts have the major responsibility for delivery of health services.

**Figure 1:**
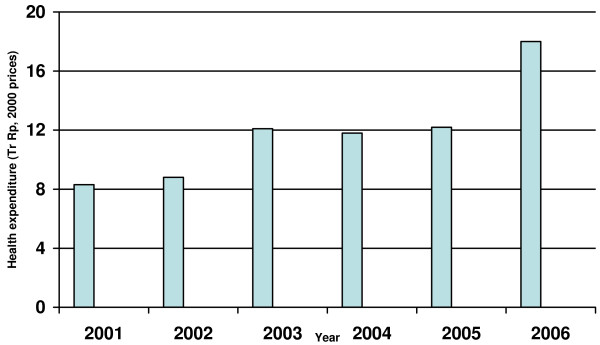
**Trend in real public health expenditure, Indonesia 2001–2006**.

Assessments of the Indonesian health system over the last decades have consistently pointed to the low levels of public funding as one of the main reasons for the disappointing performance of the sector [2-4]. For the 15 years before 2000 Indonesia spent less than 0.5% of GDP on health [3], levels considerably below those of other countries in the region [2]. Since 2000, besides the political and administrative changes, there has also been a dramatic increase in public funding of the health sector. Real public expenditure on health more than doubled between 2000 and 2006 (Figure 1), and doubled as a proportion of GDP [2].

**Figure 2:**
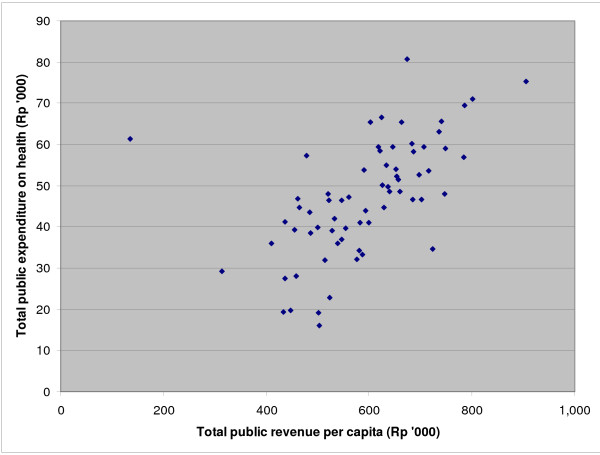
**Total district public expenditure on health per capita (routine + development) vs total district public revenue per capita, 67 districts in East, Central and West Java, 2006**.

Overall, total district public expenditure on health increases with total district revenue (Figure 2). However, this Figure also illustrates that at any given level of revenue per capita there is wide variation in per capita district expenditure on health. For example, at a public revenue level of Rp 500,000 per capita, district public expenditure on health per capita varies from less than Rp 20,000 to almost Rp 60,000, a threefold range. District governments have the discretion to allocate a portion of their revenue as they see fit and the range of expenditure on health indicates that they are making clear choices between sectors. At the same time, most districts have seen increases in public expenditure on health, even if moderate in scope in some cases.

It was widely expected that these increased funds, together with decentralization and the hope of greater freedom to change budget allocations at the district level, would lead to improvement in the delivery of services as the changed accountability relationships resulted in services more attuned to local needs. The results to date are mixed. Immunization rates remain low [5,6] and have shown little, if any improvement in the period between 2002–03 and 2006 [Heywood, P. Changes in health system performance in 10 districts in Java, Indonesia, unpublished.]; utilization of ambulatory care services is also low and self-treatment remains the most common response to illness for many [3]; there is great variation between districts in the efficiency with which resources are used [5]; and World Bank assessments across districts indicate no relationship between public expenditure on health and immunization coverage or presence of a skilled health provider at birth delivery [3]. The overall quality of care (as measured by knowledge of clinical guidelines) is low [7].

Understanding why district governments are unable to improve health services even though decentralization has provided them increased funds and, apparently, the authority to make any changes needed to do so, requires careful studies of how districts are functioning. This includes the way in which the sector is funded at the district level as well as the how those funds are allocated and utilized. Therefore this paper looks at public funds^1 ^available at the district level for health and the way in which they are used – this is important for assessment of the effects of decentralization and if we are to improve the allocation and utilization of public funds for health by districts.

Before decentralization almost all resources were transferred to district governments through centrally-specified earmarked grants. The largest of these grants covered civil service salaries and recurrent expenditures; an additional grant was used to finance development spending. Under the new system introduced in 2001 districts are still reliant on central-district transfers for more than 90% of their revenues. Although there was an early belief that earmarking would be a thing of the past^2 ^the current reality is quite different as is apparent from Tables 1 and 2. Table 1 lists and defines the various sources of funds for district government under the new system. The relative importance of the various sources for district revenues in 2005 is shown in Table 2. Aggregating across all districts and cities in the country, the most important is the General Allocation Fund (DAU) which accounts for 56% of the total. Untied funds come from own-source revenue (PAD) which contributes 9% of total, shared taxes 11%, shared natural resource revenues 12%; and tied grants under the Special Allocation Fund (DAK) 3%, and from the central line ministries, 9%. For districts on Java shared natural resource revenues are essentially zero^3^. On paper the General Allocation Fund (DAU) is untied. However, the reality is that the first call on these funds is to pay the salaries and benefits of permanent civil servants who are hired and fired by the central government but assigned to work in the districts; across the country this accounts for half the DAU. Across the country and all sectors, less than half the revenues of district governments are untied; this untied portion is made up of own-source revenue (9%), shared taxes (11%) and half the DAU (28%); the proportion untied may be even lower in some sectors and districts. This is a long way from the earlier promise of decentralization and even some of the more recent statements by, among others, the World Bank [3], which states that "....since decentralization districts decide how to spend their own resources."

**Table 1:** Public funds and flows for health sector at district level.

**Level of government**	**Source**	**Flow**	**Description**
District	Own source revenue (Pendapatan Asli Daerah – PAD)	Local revenue – included in APBD	District revenue derived from local taxes and natural resources and user charges – for health sector refers mostly to user charges at district hospital and health centers. Allocation decided by district government and approved by district parliament.
District	Shared revenues -.	Ministry of Finance, through the National Budget (APBN) to district – included in APBD	Taxes levied by the central government (especially oil, gas and personal income tax) and now shared according to an agreed formula. Sectoral allocation decided by district government and approved by district parliament.
District	General Allocation Fund (Dana Alokasi Umum – DAU)	Ministry of Finance through the National Budget (APBN) to district – included in APBD	Partially tied grant from central government direct to district government – first priority is payment of salaries of permanent civil servants, allocation of remainder decided by district government and approved by district parliament.
District	Special Allocation Fund (Dana Alokasi Khusus – DAK)	Ministry of Finance through the National Budget (APBN) to district – included in APBD	Tied grant for specific sectors from the central government direct to the district government. Can be used for construction, rehabilitation and equipment for primary care facilities. Requires 10% matching funding from the district budget.
Center	Deconcentration funds (Dekon)	Ministry of Finance to Ministry of Health (via APBN) direct to District Health Office and utilized with approval of Provincial Health Office.	Tied grants from the Ministry of Health to be used for centrally-specified sectoral activities. District proposals for use of these funds must be approved by province.
Center	Askeskin	From Ministry of Health straight to hospital or health center (with approval of District Health Office). Included in APBN.	Tied funds to cover costs of providing free healthcare to the poor.
Center	Tugas Pembantuan	Ministry of Finance direct to district hospital (with approval of MOH). Included in APBN.	Tied to physical assets, infrastructure and equipment
Center	PTT	Directly from Ministry of Finance, on recommendation of Ministry of Health, to personal account of staff member.	Tied funds for salaries and allowances for contract staff.

Source: Adapted from World Bank [3].

**Table 2:** Revenue sources for all districts and cities in Indonesia, 2005.

Source	Amount (Rp billion)	Share (%)
Own-source revenue	12,530	9
Shared taxes	15,122	11
Shared natural resource revenues	17,488	12
General Allocation Fund (DAU)	79,843	56
Special Allocation Fund (DAK)	4,628	3
Other revenue (including dekon, tugas pembantuan, PTT, Askeskin)	13,196	9

Total	142,807	100

Source: World Bank [2].

Whilst these national level figures across all sectors give a general picture, assessing the extent to which the health sector in a particular district has discretion over their public resources is difficult, made more so by the lack of accurate and detailed information on sectoral expenditure within districts. In addition, the extent of discretion may vary between different health institutions at the district level, especially hospitals as compared to health centers (HC) and the district health office (DHO)^4^. Hospitals operate quite independently of the district health office and health centers and the extent of their claim on the public budget derived from various sources might be expected to be different and vary across districts.

In essence, the public funds for health over which the district has discretion appear in the district budget for health. However, as discussed above, some of the funds in the district health budget are earmarked by the central government. The proportion so earmarked may vary between districts and could be used as an index of the potential districts have, or do not have, to vary expenditure within their total budget according to local needs.

This paper will, for a sample of districts: describe district revenue by source and basic expenditure of funds on health, including the distribution of expenditure across different health institutions and the proportion devoted to salaries; determine the extent to which districts potentially have discretion over use of the funds; and assess variation between districts.

The work reported here is part of a project to understand what is happening at the district level in the Indonesian health sector. It includes a basic enumeration of the human resources and the health facilities in which they work and deliver services as well as estimation of the financial resources available to health through the public purse at the district level. Our aim, in a sample of 15 districts in Java, is to: (i) enumerate the stock of health facilities (public and private) in the health sector in 2006; (ii) enumerate the stock of human resources (public and private) in the health sector in 2006 trained to provide care and treatment for illness – in Indonesia this means doctors, nurses and midwives; and (iii) estimate the funds (public and private) spent on health care in the course of 2006. The results will be reported in separate papers – those for health personnel and health facilities have been reported elsewhere, in [8] and [Heywood P, Harahap N: Health facilities at the district level in Indonesia, unpublished.], respectively. This paper reports on public financing for health.

## Methods

As much of the information we wished to obtain is not available from the central government we collected it in the districts. This work concentrates on Java where 60% of the Indonesian population live. Resources were sufficient to allow data to be collected in 15 districts. To ensure representation of the range of situations in Java, 5 districts were chosen in each of West Java Province, Central Java Province and East Java Province.

The 15 districts were selected as follows. Between 1997 and 2004 East Java Province and Central Java Province were included in a World Bank Safe Motherhood Project [9]. The endline data for this project were collected in 5 districts in each province (a total of 10 districts) at the time of the 2002–03 Demographic and Health Survey (DHS) [6]. The districts for the endline data collection were selected purposively by the Safe Motherhood Project team to illustrate the range of settings in which the project was implemented. The sample size in these districts was sufficient to provide district level estimates of the basic indicators in the DHS. (The DHS was repeated in the same districts, with oversampling, in 2007. A comparison of 2002–03 and 2007 DHS results for these 10 districts will be presented in a separate paper. [Heywood, P. Changes in health system performance in 10 districts in Java, Indonesia, unpublished.]) West Java was not included in the earlier Safe Motherhood Project. However, in 2007 oversampling for the DHS was carried out in 5 districts. The districts were selected purposively to illustrate the range of district settings in West Java. Table 3 shows the province, population and number of sub-districts in each of the 15 districts included in this study. Using the World Bank classification [2] all 15 districts have low fiscal revenue per capita; Cilacap and Subang have high Gross District Product per capita, the other 13 districts all have low Gross Development Product per capita.

**Table 3:** Basic information about the 15 districts included in this study.

Province	District	Population	No. Sub-districts
West Java	Ciamis	1458680	36
	Cirebon	2134656	37
	Garut	2274973	41
	Subang	1402134	22
	Sukabumi	2240901	45
Central Java	Brebes	1727708	17
	Cilacap	1717273	24
	Jepara	1078037	14
	Pemalang	1341422	14
	Rembang	591786	14
East Java	Jombang	1203716	21
	Ngawi	857449	19
	Pamekasan	782917	13
	Sampang	801541	14
	Trenggalek	682328	14

Revenue data (total and source) for the districts for 2006 were obtained from the public finance database for Indonesia (Realized Provincial and District Budget (APBD) Database) [10] maintained by the World Bank Jakarta Office using information from the Indonesian Ministry of Finance. Information from this database represents allocations, and not expenditure, to the districts.

Information on expenditure of public funds on health at the district level for 2006 was obtained from records maintained at the district health office and the district hospital. The information includes expenditure of public funds at the district level derived from the central government, provincial government and district government. The various sources of funds are defined in Table 1. The primary source of data was the financial report on expenditure^5 ^for 2006 available at the district health office and the district hospital. The primary informant in each district health office and hospital was the chief finance officer and/or the chief planning officer^6^; they were provided with a spreadsheet template (the template for data from district health office and health centers is shown as Additional File 1) for entering the data. A similar template was used to collect information for district hospitals. Further discussions with the informants were held once the initial data had been collected to, where necessary, clarify the spreadsheet entries and resolve any ambiguities.

The initial aim was to collect expenditure information using a functional classification that included salaries and remuneration, non-salary administrative activities, program activities, civil works, drugs, and equipment separately for the district health office, health centers, and hospitals. However, with the exception of information on salaries and remuneration, at least half the districts were unable to provide this level of detail for the non-salary items. Consequently, the data for each district has been summarized as in two categories, 'salaries and remuneration'^7 ^and 'other'^8 ^(see Additional File 1).

These data were collected by three teams, one for each province, in 2007. The provincial team leaders were from, and based in, the province, and had previous experience in collecting health data at the district level.

## Results and discussion

### Revenue^9^

**Figure 3:**
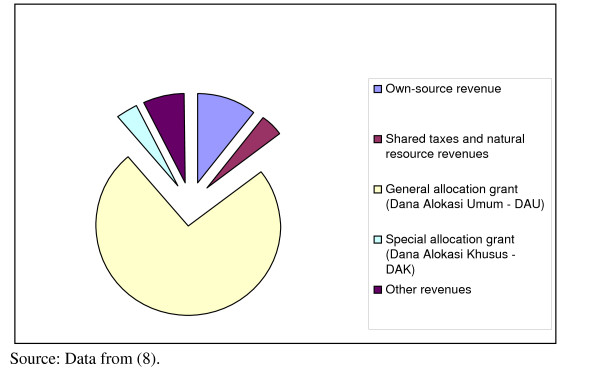
**Sources (%) of district revenue, 12 districts in East, Central and West Java, 2006**.

Nationally, real transfers to district governments almost doubled between 2000 and 2006 [2]. Over the same period real expenditure on health more than doubled (Figure 1), the largest increase occurring between 2005 and 2006. The amount and sources of district revenue for the districts in this study in 2006 are shown in Table 4 and Figure 3. The proportion contributed by the general allocation fund (DAU), 74%, is considerably higher than the 56% for the country overall (Table 2). For several of these districts the contribution of DAU reaches 80%. The higher proportion from the DAU for the districts studied here is largely due to the very low contribution of shared natural resource revenues for districts on Java (effectively zero) compared to the high levels in some other districts.

**Table 4:** Revenue by source for 12 districts in West Java, Central Java and East Java, 2006

District	Own-source revenue	Shared taxes and natural resource revenues	General allocation grant (Dana Alokasi Umum – DAU)	Special allocation grant (Dana Alokasi Khusus – DAK)	Other revenues	Total
Ciamis	55,485,790,000	24,725,680,000	708,553,000,000	52,900,000,000	64,956,000,000	906,620,470,000
Cirebon	128,170,390,000	48,320,600,000	653,606,000,000	40,910,000,000	105,759,400,000	976,766,390,000
Subang	84,102,740,000	69,667,900,000	502,000,000,000	35,360,000,000	64,178,800,000	755,309,440,000
Sukabumi	80,315,580,000	41,076,300,000	684,475,000,000	38,050,000,000	104,084,400,000	948,001,280,000
Brebes	66,203,330,000	27,032,000,000	609,557,000,000	13,850,000,000	48,028,000,000	764,670,330,000
Cilacap	109,603,600,000	27,315,000,000	609,037,000,000	0	39,277,000,000	785,232,600,000
Jepara	104,870,200,000	21,748,190,000	403,160,000,000	26,080,000,000	58,750,200,000	614,608,590,000
Pemalang	76,015,840,000	19,879,900,000	458,847,000,000	27,760,000,000	31,822,000,000	614,324,740,000
Jombang	82,688,360,000	23,087,710,000	416,553,000,000	11,210,000,000	34,174,600,000	567,713,670,000
Ngawi	27,906,620,000	15,537,772,000	450,161,000,000	25,800,000,000	24,339,826,000	543,745,218,000
Pamekasan	41,001,839,000	15,962,000,000	373,618,000,000	26,130,000,000	19,295,320,000	476,007,159,000
Sampang	26,190,020,000	18,275,000,000	330,911,000,000	30,090,000,000	10,315,000,000	415,781,020,000
Total	882,554,309,000	352,628,052,000	6,200,478,000,000	328,140,000,000	604,980,546,000	8,368,780,907,000
Percent 12 districts	11	4	74	4	7	100

Source: World Bank public expenditure in Indonesia database [10]

### Level of public spending on health at the district level

Across the 15 districts studied the overall level of public expenditure for health at the district level is Rp 59,080 per person per year. This is consistent with the per capita estimate for the whole country derived from budget data available at the national level – this macro approach yields an estimate of Rp 62,332 per person per year at the district level (Table 5).

**Table 5:** Public expenditures on health by level of government, 2006

Level of government	Total	Per capita	
		
	Rp (billions)	Rp (thousands)	%
Central	12,190	54,664	39
Province	5,100	22,870	16
District	13,900	62,332	45

**Total**	**31,190**	**139,865**	**100**

Source: World Bank [3]; Authors' calculations. Population 2006 assumed to be 223 million.

There is wide variation between districts in per capita expenditure of public funds on health – for these 15 districts the range is from Rp 39,873 in Cilacap to Rp 104,355 in Pamekasan – see Tables 6, 7 and 8 which summarize the main variables by district for West Java, Central Java and East Java, respectively.

**Table 6:** Expenditure (Rupiah) of public funds in health sector of 5 districts in West Java Province by source of funds, 2006. (Note: 1 US $ is equal to approximately Rp. 10,000).

District			Source of public expenditure				Total
				
			District government	Provincial government	Central government	Other	
Ciamis	Total	Rp.	19,320,039,814	6,398,592,632	50,010,761,286	7,479,602,817	83,208,996,549
	Per Capita	Rp.	13,245	4,387	34,285	5,128	57,044
		%	23	8	60	9	100
Cirebon	Total	Rp.	79,387,460,213	222,000,000	61,472,764,207	8,489,849,200	149,572,073,620
	Per Capita	Rp.	37,190	104	28,798	3,977	70,068
		%	53	0	41	6	100
Garut	Total	Rp.	55,769,315,362	2,855,756,600	60,566,083,380	2,588,750,000	121,779,905,342
	Per Capita	Rp.	24,514	1,255	26,623	1,138	53,530
		%	46	2	50	2	100
Subang	Total	Rp.	35,428,169,696	5,255,488,300	44,309,003,130	2,293,684,978	87,286,346,104
	Per Capita	Rp.	25,595	3,797	32,011	1,657	63,060
		%	41	6	51	3	100
Sukabumi	Total	Rp.	43,832,905,927	4,235,468,802	39,981,841,157	2,798,425,000	90,848,640,886
	Per Capita	Rp.	19,560	1,890	17,842	1,249	40,541
		%	48	5	44	3	100
5 districts	Total	Rp.	233,737,891,012	18,967,306,334	256,340,453,160	23,650,311,995	532,695,962,501
	Per Capita	Rp.	24,621	1,998	27,002	2,491	56,112
		%	44	4	48	4	100

Source: Authors' calculations.

Note: Funds included in 'Other' are Loans and Grants.

**Table 7:** Expenditure (Rupiah) of public funds in health sector of 5 districts in Central Java Province by source of funds, 2006. (Note: 1 US $ is equal to approximately Rp. 10,000).

District			Source of public expenditure				Total
				
			District government	Provincial government	Central government	Other	
Brebes	Total	Rp.	31,176,108,000	18,040,000	38,745,942,000	117,543,000	70,057,633,000
	Per capita	Rp.	18,045	10	22,426	68	40,549
		%	45	0	55	0	100
Cilacap	Total	Rp.	41,032,210,000	0	27,439,830,000	0	68,472,040,000
	Per capita	Rp.	23,894	0	15,979	0	39,873
		%	60	0	40	0	100
Jepara	Total	Rp.	39,394,861,000	549,720,000	31,414,180,000	0	71,358,761,000
	Per capita	Rp.	36,543	510	29,140	0	66,193
		%	55	1	44	0	100
Pemalang	Total	Rp.	44,257,660,000	0	38,737,409,000	0	82,995,069,000
	Per capita	Rp.	32,993	0	28,878	0	61,871
		%	53	0	47	0	100
Rembang	Total	Rp.	37,603,767,000	74,000,000	21,706,242,000	216,335,000	59,600,344,000
	Per capita	Rp	63,543	125	36,679	366	100,713
		%	63	0	36	0	100
5 districts	Total	Rp.	193,464,606,000	641,760,000	158,043,603,000	333,878,000	352,483,847,000
	Per capita	Rp	29,966	99	24,479	52	54,596
		%	55	0	45	0	100

Source: Authors' calculations.

Note: Funds included in 'Other' are Loans and Grants which were low in this province in 2006.

**Table 8:** Expenditure (Rupiah) of public funds in health sector of 5 districts in East Java Province by source of funds, 2006. (Note: 1 US $ is equal to approximately Rp. 10,000)

District			Source of public expenditure				Total
				
			District government	Provincial government	Central government	Other	
Jombang	Total	Rp.	44,927,109,165	0	39,827,901,347	1,424,756,412	86,179,766,924
	Per capita	Rp.	37,324	0	33,087	1,184	71,595
		%	52	0	46	2	100
Ngawi	Total	Rp.	12,834,992,053	0	40,267,935,189	224,420,000	53,327,347,242
	Per capita	Rp.	14,969	0	46,962	262	62,193
		%	24	0	76	0	100
Pamekasan	Total	Rp.	28,182,851,555	695,799,500	49,048,028,942	3,774,751,000	81,701,430,997
	Per capita	Rp.	35,997	889	62,648	4,821	104,355
		%	34	1	60	5	100
Sampang	Total	Rp.	12,618,332,276	495,001,500	24,709,504,397	2,394,618,295	40,217,456,468
	Per capita	Rp.	15,743	618	30,827	2,988	50,175
		%	31	1	61	6	100
Trenggalek	Total	Rp.	19,477,396,309	0	31,873,194,060	50,270,000	51,400,860,369
	Per capita	Rp.	28,546	0	46,712	74	75,332
		%	38	0	62	0	100
5 districts	Total	Rp.	118,040,681,358	1,190,801,000	185,726,563,935	7,868,815,707	312,826,862,000
	Per capita	Rp.	27,274	275	42,913	1,818	72,281
		%	38	0	59	3	100
15 districts	Total	Rp.	545,243,178,370	20,799,867,334	600,110,620,095	31,853,005,702	1,198,006,671,500
	Per capita	Rp.	26,889	1,026	29,595	1,571	59,080
		%	46	2	50	3	100

Source: Authors' calculations.

Note: Funds included in 'Other' are Loans and Grants

The broader context for these district level public funds is that they represent 45% (Table 4) of the total health expenditure from public sources – for the remainder, 16% of overall health expenditure is at the province level and 39% at the central level. Overall, then, the additional expenditures at the province and central levels represent more than Rp 70,000 per person per year in 2006. The district level expenditures are primarily for the delivery of publicly funded services while much of the central and provincial level funds are for activities far removed from, and which contribute little to, service delivery.

### Sources of public funds for health at the district level

Taking the district as a whole, public financing derives from three government sources – district government, provincial government and central government. The shares of these three sources are shown in Tables 6, 7 and 8, (which show the shares for the 5 districts in West Java, Central Java and East Java, respectively). A summary across the 15 districts is shown in Table 9. For the sector as a whole, across these 15 districts, the central government provides a little more than half the public funds while the district government provides just over 40%. The provincial government is not an important source of funds for the districts and loans and grants account for less than 5%. The variation in these shares between districts is wide. Thus, the share of district government varies from a low of 23% in Ciamis to a high of 63% in Rembang. The contribution of central government varies from a low of 36% in Rembang to a high of 76% in Ngawi.

**Table 9:** Source of public funds (%) for District health office, health centers and district hospitals across 15 districts in West and East Java Provinces, 2006.

Source	District health office (%)	Health Centers (%)	District hospitals (%)	Total (%)
District government	47	24	61	42
Provincial government	2	4	1	2
Central government	42	67	38	52
Other (Loans/grants)	10	6	0	4

Total	100	100	100	100

Source: Authors' calculations.

### How are the public funds for health at the district level spent?

The public funds at the district level are spent through three health institutions – the district health office, health centers, and the district public hospital. As the district health office and health centers essentially operate as an administrative and financial unit^10 ^they are combined in this analysis and referred to as DHO/HC. Across all 15 districts the DHO/HC accounts for 57% of expenditure and district hospitals for 43% (see Table 10). Again, there is considerable variation between districts with the proportion of district health expenditure accounted for by hospitals varying from 24% in Ciamis to 64% in Jombang.

**Table 10:** Expenditure (Rupiah) and share (%) of public funds on the district health office + health centers and district hospitals in 15 districts of West, Central and East Java Provinces, 2006.

Province	District	District health office and health centers		District hospitals	
		Rupiah	Percent	Rupiah	Percent
West	Ciamis	63,628,626,591	76	19,580,369,958	24
Java	Cirebon	84,575,424,038	57	64,996,649,582	43
	Garut	65,970,093,460	54	55,809,811,882	46
	Subang	53,934,449,208	62	33,351,896,896	38
	Sukabumi	53,565,197,905	59	37,283,442,981	41
Central	Brebes	52,233,671,229	75	17,823,961,892	25
Java	Cilacap	33,316,917,801	49	35,155,122,798	51
	Jepara	32,230,879,000	45	39,127,881,501	55
	Pemalang	47,620,495,537	57	35,374,273,486	43
	Rembang	26,869,962,328	45	32,730,382,190	55
East	Jombang	31,039,475,155	36	55,140,291,769	64
Java	Ngawi	32,910,591,706	62	20,416,755,536	38
	Pamekasan	39,382,283,562	48	42,319,147,435	52
	Sampang	27,976,333,567	70	12,241,122,901	30
	Trenggalek	36,455,528,470	71	14,945,331,899	29
					
	Total for 15 districts	681,709,929,557	57	516,296,442,705	43

Source: Authors' calculations.

(Note: 1 US $ is equal to approximately Rp. 10,000)

There is little reliable information at the district level on which to base an analysis of expenditure by function. Current attempts to develop a system of national health accounts use budget allocations rather than expenditures and have so far not adopted a standardized functional classification of expenditures by function that would allow comparison across studies and with other countries. Further, and partly as a result of the failure to develop a standardized system of accounts in the health sector, there is considerable variation between districts in allocation of particular expenditures to specific activities; as a result it is not possible, so far, to make an accurate functional analysis of expenditure. However, we do know that the largest single item of expenditure at the hospital, health center and district health office level is for salaries and remuneration^11^. The proportion of public funds from district and central government, and the overall total, expended on salaries and remuneration by district is shown in Table 11. Perhaps the most noteworthy aspect of this table is the relative uniformity of the proportion of the total expended on this function – it ranges from the 32% in Sukabumi and Sampang to 46% in Ciamis and Jepara. Across all 15 districts the proportion of the total expenditure on salaries and remuneration is 40%.

**Table 11:** Remuneration as a proportion (%) of health expenditure by district and central governments and total district expenditure for health.

District	District government	Central government	Total health expenditure
Subang	30	54	40
Garut	26	61	42
Ciamis	16	70	46
Cirebon	25	58	37
Sukabumi	19	51	32
Brebes	24	59	43
Jepara	32	66	46
Cilacap	43	47	45
Pemalang	16	54	34
Rembang	33	65	44
Ngawi	12	44	36
Jombang	42	46	43
Pamekasan	51	37	40
Sampang	45	29	32
Trenggalek	34	44	41
**15 districts**	**30**	**53**	**40**

Source: Authors' calculations.

The proportion of funds for the DHO/HC and hospitals at district level is shown in Table 12. Within this relatively narrow range for the total there is great variation in the proportion of salaries and remuneration contributed by the district and central governments to the DHO/HC (overall 16% from district and 84% from central funds) and to hospitals (57% from district and 43% from the center). In 2006, across these 15 districts we can summarize the following with respect to salaries and remuneration:

**Table 12:** Proportion of salaries derived from central government (APBN) and district government (APBD) expenditure by health institution and district. 2006

District	DHO/HC		Hospital		Total	
	APBD	APBN	APBD	APBN	APBD	APBN
Subang	0.09	0.91	0.63	0.37	0.31	0.69
Garut	0.03	0.97	0.67	0.33	0.28	0.72
Ciamis	0.02	0.98	0.31	0.69	0.08	0.92
Cirebon	0.11	0.89	0.62	0.38	0.36	0.64
Sukabumi	0.00	1.00	0.55	0.45	0.29	0.71
Brebes	0.16	0.84	0.53	0.47	0.25	0.75
Jepara	0.37	0.63	0.38	0.62	0.38	0.62
Cilacap	0.77	0.32	0.38	0.62	0.58	0.42
Pemalang	0.03	0.97	0.55	0.45	0.25	0.75
Rembang	0.12	0.88	0.71	0.29	0.47	0.53
Jombang	0.29	0.71	0.74	0.26	0.51	0.49
Ngawi	0.00	1.00	0.65	0.35	0.08	0.92
Pamekasan	0.29	0.71	0.51	0.49	0.44	0.56
Sampang	0.37	0.63	0.61	0.39	0.44	0.56
Trenggalek	0.26	0.74	0.46	0.54	0.32	0.68
15 districts	0.16	0.84	0.57	0.43	0.34	0.66

Source: Authors' calculations.

Note: DHO/HC = District health office and health centers. APBD = district health expenditures using district funds. APBN = district health expenditures utilizing national funds.

• Salaries and remuneration account for 40% of all public expenditure for health at the district level, with considerable variation between districts in the proportion of salaries contributed by central and district governments (Table 11).

• Funds from central government account for 66% (see Table 12) of the total funds for salaries and remuneration – the staff for whom the central government pays are predominantly permanent civil servants; funds from the district government account for the remaining 34% – the staff for whom the district government pays are predominantly not permanent civil servants.

• Central government funds account for 52% (see Table 9) of all public expenditure on health in the district; of these central government funds, 53% is for salaries and remuneration (see Table 11).

• A prominent feature of the results is the considerable variation between districts on almost all funding parameters.

Overall, the proportion of salaries and remuneration accounted for by the central government will have risen during 2007 and 2008 as many contract staff (mostly paid for by the district) took up the offer to convert to permanent civil servant status and will now be paid for from the DAU.

### District discretion over use of district level public funds for health

**Figure 4:**
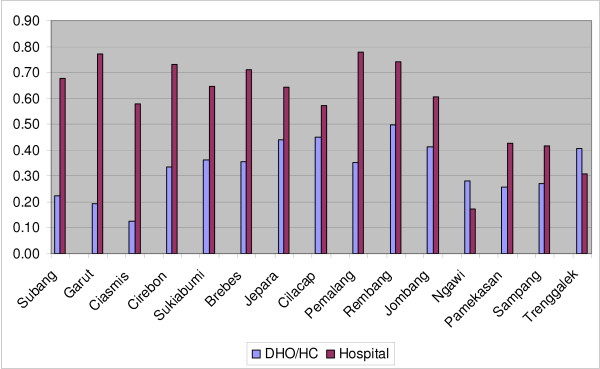
**Proportion of health spending in District Health Office/Health Center and District Hospital over which district can potentially exercise discretion**.

An important issue in the context of decentralization is the extent to which districts have discretion over the use of the public funds for health spent in the district. District governments have no control over tied central government funds (see Table 1 for details) allocated to specific expenditures. For the DAU, salaries receive first preference and the district has little control. Similarly the districts do not have control over the tied grants from the center through DAK, deconcentration and tugas pembantuan^12^, or the other resources used to reimburse health facilities for services to the poor and to public servants, and from loans and grants which specify the way in which the funds are to be used. Thus, an index of the extent to which the districts can potentially have discretion over the funds expended for health at the district level is to determine the proportion that remains after subtracting funds that are "centrally controlled"^13^. This is shown in Table 13 and Figure 4. The important point here is that whilst overall the proportion over which a district could potentially exercise discretion varies from 23% in Ciamis to 63% in Rembang (but overall is a seemingly reasonable 45%), these figures hide a wide and systematic difference between DHO/HC and hospitals. With only two exceptions (Ngawi and Trenggalek) hospitals have much greater potential for discretion in use of their funds (63% overall) than do the DHO/HC (31% overall). In general this correlates with the proportion of salaries derived from the central government (Table 12) and user charges at the hospitals – the lower the proportion from the central government the greater the contribution from user charges, the higher the potential for discretion in use of funds; and as the proportion of salaries is highest for DHO/HC the funds over which they potentially have discretion are much lower.

**Table 13:** Proportion of public sector health expenditure in district over which district can possibly can exercise discretion

	DHO/HC	Hospital	Total
Subang	0.22	0.68	0.40
Garut	0.19	0.77	0.46
Ciamis	0.13	0.58	0.23
Cirebon	0.34	0.73	0.51
Sukabumi	0.36	0.64	0.48
Brebes	0.35	0.71	0.45
Jepara	0.44	0.64	0.55
Cilacap	0.45	0.57	0.51
Pemalang	0.35	0.78	0.53
Rembang	0.50	0.74	0.63
Jombang	0.41	0.61	0.54
Ngawi	0.28	0.17	0.24
Pamekasan	0.26	0.43	0.34
Sampang	0.27	0.42	0.31
Trenggalek	0.41	0.31	0.38
			
15 districts	0.31	0.63	0.45

Source: Authors' calculations.

Note: the proportion of funds over which the district could potentially have control is equal to 1.00 minus the proportion of centrally controlled funds (DAU for salaries, DAK, Dekon, Tugas pembantuan, Askeskin, Askes PNS, loans and grants). These various funds are defined in Table 1.

Even though the data are summarized at the district level, a unit with meaning within the Indonesian administrative system, there is no overall view of the sector at that level because it is fractured along at least four dimensions or fault lines, all of which reflect funding in some way.

• Public/private – two-thirds of the funds expended in the sector come from the private sector and one-third from the public sector [3];

• District/center – only about half of all public funds for health are spent at the district level (see Table 4);

• DHO/HC and hospital – within the district, public funds for health are effectively divided between two institutions, the DHO/HC and the district hospital, between which there is no coordination, they are funded separately and administered separately (Table 10);

• Discretion/no discretion – across the 15 districts less than half the public funds (Table 13) expended for health at the district level are controlled by the district government – in two districts it is less than one-quarter^14^; the other half is controlled by the central government which effectively dictates the way in which they are used. Further, there are major differences between DHO/HC and hospitals in the potential for discretion in use of funds – the potential is much greater in hospitals than in DHO/HC.

To summarize: on paper, of the public funds spent in the district on health about half (Table 13) are apparently controlled by the district, but these are divided between the DHO/HC and the hospital. Moreover, some of these apparently discretionary funds must be spent on inputs that complement the fixed costs represented by the salaries – medical consumables and utilities in particular. The result is that there is limited discretion at the district level on use of public funds for health, a conclusion consistent with that reached by others [2]; the proportion overall is probably less than one-third and would be much lower for DHO/HC^15^. The room for innovation and change by the public sector to address local problems is very limited and will become even more so if the government continues to increase the number of permanent public servants in the sector, thereby reducing the proportion of public funds at the district level over which the district government could potentially exercise discretion.

Apart from the limited control by the district government over use of public funds for health, there are, in effect, no mechanisms which enable taking a view of the sector as a whole at any level, and especially at the district level. Central control over salaries, the Special Allocation Fund, the deconcentration and tugas pembantuan funds, all mean that individual institutions and district administrations react to single lines of funding from the center; no one has an incentive to view the sector as a whole. At the same time there are perverse incentives for the district in the current funding formulas – full coverage by the central government of the wage bill for permanent civil servants at the district level provides a disincentive for district governments to streamline and modernize their civil services [2]. Although there is a strong case for reform of these tied funds to allow district administrations the flexibility to respond to the local health problems and incentives to introduce reforms, strong opposition from vested interests in the central government has so far managed to delay any reforms.

For many years the constant excuse given for the mediocre performance of the Indonesian health system was that the public funds allocated to it were just too few, it was not possible (so it was argued) to improve services without a major increase in funds and that the straight-jacket of central control and one-size-fits-all precluded local level responses to local situations. The increased funding that came with decentralization is, in one way, a robust response to these claims. Between 2000 and 2006 the central government funds transferred to district governments almost doubled in real terms. Over the same period expenditure on health as a proportion of GDP went from 0.50% to 0.95% [2]. Yet there is great variation between districts in the efficiency with which public resources are used [5], no relation between health system output and public expenditure [3], and there seems to have been little improvement in performance of the health system^16 ^with the increased public funds and decentralization, begging the question as to why this has been the case?

Part of the answer lies in the fact that the overall public system in place today is much the same as the one in place before decentralization. And this is a system that was not designed to permit, let alone encourage, local level decision making. The system was designed to implement central decisions, not local ones. There is still a tendency, encouraged and reinforced by the central ministry, for the districts to look to the center on major issues – until this changes progress at the district level will be slow.

Part of the answer to the question, as indicated by this study and other analyses of the sector as a whole [2], is that there has been, in effect, very little decentralization in the health sector, in the sense that the district is now the place where the decisions about the use of public money are made. Far from it. Most of the critical decisions are still made by the central government. The central government hires and pays permanent civil servants, a move that allows it to control the overall direction of the service and which, at the same time provides a disincentive for districts to reform the sector – why attempt to improve the low productivity of the sector staff when the central government will continue to pay? Apart from paying for, and eventually controlling, the most important resource in the sector, the central government also controls critical sector development through decisions about other tied sources of funding. In that sense little has changed. It is true that districts are now more autonomous in that they no longer respond to orders from the center in the ways they did during the Suharto era. It is true that the role of the province has been greatly reduced and that districts can now ignore the province if they wish. It is true that the central government continues to issue new laws and regulations on decentralization^17^. But for all intents and purposes key decisions about money are still made by the central government, and no one is held accountable for the performance of the sector – the district blames the center and the central ministries (and their ministers) are not accountable to district populations.

What the sector needs now is some real decentralization, such that the district government has real discretion over, say, 75% of the public funds in the health sector, including funds for salaries, and is held accountable through transparent agreements with the central government for the sector performance. Under the present arrangement the district can always blame the center for its performance – levels of funding are not sufficient, staff levels and salaries are decisions made by the central government, it is difficult to plan when the central government retains so much control.

For districts to be more accountable, the sector needs to change in such a way that the fracture lines are reduced. Two-thirds of funding for health is private so the sector needs to be managed as a whole; it is the districts where services are delivered, districts governments are responsible for the health services and need discretion over most of the public funds for health; health centers and hospitals are part of an overall health system and districts need to manage and coordinate the services they offer in conjunction with those available through the private sector. And all of this is occurring in the context of the relentless pressure from the epidemiological and demographic transition and the consequent changes in the amount and type of care required.

There are two pre-requisites for greater coordination of the sector as a whole at the district level – central government control needs to be at a minimum and that means allowing, even encouraging, the district to make decisions about the sector. That in turn means that the district needs to develop a long term view of where the health sector is going and how it is going to get there. The district has never been asked to do that before so it is not surprising that the skills and background to do so are not present at the moment. There is clearly a role for the center here – to provide leadership which sets goals and broad guidelines for the sector, promotes local approaches to local problems and introduces incentives for districts (and the provinces) to collaborate on matters where economies of scale and external effects should be addressed. This can best be approached by controlling a limited proportion (no more than 25%) of the public expenditure and using it in a strategic manner to provide incentives for districts to achieve a limited number of national objectives and priorities. So far the center is not providing that leadership either. In fact, the goal seems to be to re-gain as much central control as possible, a goal that, in effect turns back the clock on decentralization in a vast and diversified country.

Finally, this paper has shown that there is a lot of variation between districts on most of the variables examined. There is, likewise, considerable variation in the human resources for health [8] and health facilities [8,11] [Heywood P, Harahap N: Health facilities at the district level in Indonesia, unpublished.] in these same districts and in the effectiveness with which public funds are used. What is important now is examining why these differences have arisen and how they are influencing delivery of services, health care and health outcomes, and the implications for the health system in the future.

## Conclusion

During the Suharto era public funding of health in Indonesia was low and the health services were tightly controlled by the central government; district health staff had practically no discretion over expenditure. Following the downfall of President Suharto there was a radical political, administrative and fiscal decentralization with delivery of services becoming the responsibility of district governments. In addition, public funding for health services more than doubled between 2001 and 2006. It was widely expected that services would improve as district governments now had more adequate funds and the responsibility for services. To date there has been little improvement in services. Understanding why services have not improved requires careful study of what is happening at the district level. The public expenditure information collected in 15 districts as part of this study indicates district governments are reliant on the central government for as much as 90% of their revenue; that approximately half public expenditure on health is at the district level; that at least 40% of district level public expenditure on health is for personnel, almost all of them permanent civil servants; and that districts have discretion over less than one-third of district public expenditure on health; the extent of discretion over spending is much higher in district hospitals; in contrast, district health offices and health centers have discretion over the use of less than a quarter of public funds for health. The current trend is for the proportion of public funds over which the districts have discretion to decrease. There is considerable variation between districts. In contradiction to the promise of decentralization there has been little increase in the potential for discretion at the district level in managing public funds for health – this is likely to be an important reason for the lack of improvement in publicly funded health services. Key decisions about money are still made by the central government, and no one is held accountable for the performance of the sector – the district blames the center and the central ministries (and their ministers) are not accountable to district populations.

## Competing interests

The authors declare that they have no competing interests.

## Authors' contributions

PH conceived the study, analyzed results and drafted the manuscript. NPH provided input on study design, supervised data collection in West Java Province, assisted with interpretation of results, and reviewed manuscript. Both authors read and approved the final manuscript.

## Appendix 1. Endnotes

^1 ^The largest component of public spending on health in Indonesia is undertaken through the Ministry of Health (MOH) and related arms of government at the provincial and district levels. Although other government departments also make some expenditures related to health, the total amounts are small relative to that of the MOH and are not included in the funds discussed in this paper.

^2 ^See, for example, World Bank [1] which states "......In the new system, central-regional transfers remain the dominant means of financing, **but the earmarking is gone**." (emphasis added).

^3 ^Revenues from natural resources are concentrated in oil producing regions, none of which are on the island of Java [2].

^4 ^Within a district, funds to the DHO and HCs are through a single budget document proposed by the District Health Office, reviewed by the District Planning Office and approved by the district parliament. The HCs are administratively responsible to the DHO. In contrast, the district hospital(s) reports directly to the Head of the District and has a separate budget document which is proposed and approved separately from that for the DHO/HC.

^5 ^The actual name of this document varies between districts and health institutions but all responded readily to our requests for the financial report on expenditure for 2006.

^6 ^The person most familiar with the financial report for 2006 also varied between districts and health institutions. In most hospitals it was the chief budget officer while in district health offices it was usually, but not always, the chief planning officer.

^7 ^'Salaries and remuneration' expenditures are defined as including: salaries for all staff in the institution (district health office, health center, hospital; fee-for-service distributed to staff; overtime; honoraria (for projects, training, special activities, seminars, workshops); civil service allowances (e.g. food and welfare allowances).

^8 ^'Other' refers to expenditures apart from salaries and remuneration and includes expenditures for: non-salary administrative activities, program activities, civil works, drugs, and equipment.

^9 ^The work of which this report forms a part looks at 15 districts on Java. However, the public finance database maintained by the World Bank has complete data for only 12 of these districts and it is those districts discussed in this section.

^10 ^See Endnote 4.

^11 ^See Endnote 7 for definition.

^12 ^See Table 1 for a definition of these various sources of funds.

^13 ^Thus, the proportion of funds over which the district potentially has control is equal to 1.00 minus the proportion of centrally controlled funds (DAU for salaries, DAK, Dekon, Tugas pembantuan, Askeskin, Askes PNS, loans and grants). These various funds are defined in Table 1.

^14 ^In both these districts – Ciamis and Ngawi – the proportion of all salaries accounted for by the central government is over 90%.

^15 ^The broader context is that only one-third of the funds expended in the health sector are public [3]; about half the public funds for health (from central, provincial and district sources) are spent at the district level (Table 4); and then no more than one-third of the public funds at the district level are discretionary. Thus, discretionary public sector funds for health at the district level represent as little as one-eighteenth (1/3 × 1/2 × 1/3) (approximately 5%) of total health expenditure.

^16 ^Changes in performance of the health system between 2002–03 and 2007 in the 10 districts in East Java and Central Java included in this study will be addressed in [Heywood, P. Changes in health system performance in 10 districts in Java, Indonesia, unpublished.]. The study will use a comparison between the results of the Demographic and Health Survey (DHS) in these years when over sampling in the 10 districts allowed estimates of selected health indicators at the district level.

^17 ^For example, Law 32 in 2004 and Law 33 in 2004 as well as government regulation (PP) Number 7 in 2008.

## Supplementary Material

Additional File 1 **Additional Table 1. Public spending on health (realization) at the DISTRICT HEALTH OFFICE and PUSKESMAS by source and expenditure category 2006 financial year**. Table showing template for collection of public expenditure on health at the district level. Click here for file

## References

[B1] World Bank (2003). Decentralizing Indonesia: a regional public expenditure review – overview report. Report No 26191-IND.

[B2] World Bank (2007). Spending for development: making the most of Indonesia's new opportunities. Indonesia Public Expenditure Review 2007.

[B3] World Bank (2008). Investing in Indonesia's health: challenges and opportunities for future public spending.

[B4] Aparnaa Somanathan, Ravindra Rannan-Eliya, Tharanga Fernando (2004). Indonesia Public Health Expenditure Review. Institute of Policy Studies – Health Policy Programme Colombo Sri Lanka.

[B5] Indonesia NIHRD (2005). Indonesia: sub-national health system performance assessment.

[B6] BPS-Statistics Indonesia (2003). Indonesia Demographic and Health Survey 2002–2003.

[B7] Barber SL, Gertler PJ, Harimurti P (2007). Differences in access to high-quality outpatient care in Indonesia. Health Affairs.

[B8] Heywood P, Harahap NP (2009). Human resources for health at the district level in Indonesia: the smoke and mirrors of decentralization. Human Resources for Health.

[B9] World Bank (1997). Safe Motherhood Project: a partnership and family approach. Project Appraisal Document.

[B10] World Bank Public expenditure in Indonesia database. http://www.worldbank.org/id/publicexpenditure.

[B11] Heywood P, Harahap NP (2008). Health facilities at the district level in Indonesia. Submitted to Health Servcies Research.

