# Supplementary material for: Public funding of health at the district level in Indonesia after decentralization – sources, flows and contradictions
Source: Health Res Policy Syst. 2009 Apr 16;7:5. doi: 10.1186/1478-4505-7-5 (PMC2678112; doi:10.1186/1478-4505-7-5)
Supplement: Additional File 1 — Additional Table 1. Public spending on health (realization) at the DISTRICT HEALTH OFFICE and PUSKESMAS by source and expenditure category 2006 financial year. Table showing template for collection of public expenditure on health at the district level. [file 1478-4505-7-5-S1.pdf]

Additional Table 1. Public spending on health (realization) at the DISTRICT HEALTH OFFICE and PUSKESMAS by source and expenditure category 2006 financial year

Source of funds	Salaries and remuneration			Non-salaries and remuneration			Totals		TOTAL
	DHO	HC	Total	DHO	HC	Total	Dinas	Pusk.	
**I. NATIONAL BUDGET**									
**1. General Allocation Fund (DAK)**									
**2. Tied funds (Tugas Pembantuan)**									
Construction, Equipment, Vehicles									
**3. Deconcentration funds**									
National immunization week									
Maternal health									
Child health									
Basic health care									
Health management and policy									
Nutrition									
Health promotion and community empowerment									
Control of food, drug and dangerous substances									
**4. Salaries and remuneration**									
Permanent civil servants									
Contract									
Honoraria									
**5. Health insurance for the poor**									
**II. PROVINCIAL BUDGET**									
**1. Salaries and remuneration**									
Contract									
Honoraria									
**2. Program**									
**3. Governor's assistance**									
**III. DISTRICT**									
**1. Salaries**									
Contract									
Fee-for-service									
Honoraria									
**2. Routine non-salary**									
**3. Program**									
**IV. Loans and grants**									
**TOTAL**									
